# Hearing voices and other altered perceptual experiences across psychotic, mood, and anxiety disorders: from phenomenology and mechanisms to future directions

**DOI:** 10.1038/s41537-025-00673-3

**Published:** 2025-09-29

**Authors:** Wei Lin Toh, Sophie Richards, Charles Fernyhough, Eleanor Longden, Peter Moseley, Padmavati Ramachandran, Neil Thomas, Susan Lee Rossell

**Affiliations:** 1 Centre for Mental Health & Brain Sciences, Swinburne University of Technology, Melbourne, VIC Australia; 2 Department of Psychology, Alfred Hospital, Melbourne, VIC Australia; 3 Department of Psychiatry, St Vincent’s Hospital, Melbourne, VIC Australia; 4 Department of Psychology, Durham University, Durham, UK; 5 Psychosis Research Unit, Greater Manchester Mental Health NHS Foundation Trust, Manchester, UK; 6 Division of Psychology & Mental Health, School of Health Sciences, Faculty of Biology, Medicine & Health, Manchester Academic Health Science Centre, University of Manchester, Manchester, UK; 7 Complex Trauma & Resilience Research Unit, Greater Manchester Mental Health NHS Foundation Trust, Manchester, UK; 8 Department of Psychology, Northumbria University, Newcastle-upon-Tyne, UK; 9 Schizophrenia Research Foundation, Chennai, India

**Keywords:** Psychosis, Schizophrenia

## Abstract

While voice-hearing in psychosis has received much attention, perceptual experiences in other sensory modalities and psychiatric conditions have remained relatively overlooked. The present review aimed to address this gap by providing an overview of voices/altered perceptual experiences (APE) across psychotic, mood and anxiety disorders in terms of phenomenological characteristics, biopsychosocial mechanisms, etiological models and therapeutic interventions. Where possible, lived experience perspectives and transcultural considerations were embedded. A narrative literature review was conducted. Knowledge pertaining to voices in psychosis formed the foundation, broadened to include other sensory modalities and diagnostic conditions. Quality assessment demonstrated an excellent rating of 12/12. Notable findings related to: (i) phenomenological heterogeneity in voices/APE within individuals and across diagnostic conditions, with multisensory/multimodal experiences relatively widespread; (ii) existing mechanistic studies mainly focusing on the role of trauma and neurocognition in voices; (iii) prevailing explanatory models mostly focusing on voices; (iv) a need for emerging interventions to extrapolate to encompass broader therapeutic applications; and (v) wide-ranging specificity issues and transcultural considerations to be addressed. Future research should invest in appropriate assessment tools as well as ensuring methodological consistency in mechanistic studies. Incorporating lived experience perspectives and meaningfully embedding transcultural considerations in theoretical and empirical ways are also essential.

## Introduction

Voice-hearing and other atypical perceptual experiences represent a profound philosophical and psychiatric conundrum that has fascinated scholars and carried significant meaning for affected individuals. For some people, these experiences may be associated with disability, distress, and risk of self-harm^1^, for which existing treatments are only moderately effective^2^. Our understanding to date has largely focused on what has been perceived as its most common manifestation—hearing voices^3^. However, such experiences are also prevalent in other (and at times, multiple) sensory modalities^4–7^. A thorough understanding of these phenomena has notable implications for scientific knowledge, clinical practice, and therapeutic advancement. By comprehensively amalgamating and reviewing existing literature and theories, it may be possible to understand where more focused systematic reviews, as well as targeted empirical studies, are needed, in addition to highlighting potential empirical and clinical gaps in the existing literature.

### Definitions and terminology

‘Hearing voices’ is defined as the perception of auditory events, commonly in the form of heard speech, in the absence of corresponding external stimuli. Atypical perceptual experiences can also occur across other senses, including visual (i.e., seeing images), somatic-tactile (i.e., feeling sensations on or under the skin), olfactory (i.e., smelling odours), and gustatory (i.e., tasting flavours) modalities. Together, these experiences are termed ‘hallucinations’. This phrase may carry certain medical or social connotations that some may consider marginalising, e.g., ref. ^8^, whereas others may welcome a biological explanation for their experiences. In this literature review, we will preference ‘voices/altered perceptual experiences (APE)’ in line with lived experience input, mostly employing ‘hallucinations’ to refer to experiences more collectively and taking care to denote the sensory modality under study. Alongside unimodal (i.e., a single modality) phenomena, multimodal and multisensory hallucinations are likewise noteworthy^6^. Many people will have APE across two or more sensory modalities, with experiences that co-occur in time and/or are related thematically referred to as ‘multimodal’ in the literature^9^ (although consensus has yet to be reached on exact operationalisations.)

### Scope and research questions

This literature review focuses on examining voices/APE as a transdiagnostic target across intersecting psychiatric conditions involving psychosis, mood and anxiety. This is supported by existing syntheses that have established voices/APE as occurring across a range of diagnostic conditions^10^, and aligns with approaches seeking to understand mental illness on a symptom level^11^. Attention was centred on psychosis, mood and anxiety (neurological disorders where most work on visual hallucinations has derived was also briefly touched on) and, where applicable, the general population. Adults formed the focus, with child and adolescent experiences acknowledged when appropriate. Notably, specificity issues exist regarding conflation amongst levels of diagnosis (i.e., schizophrenia), syndrome (i.e., positive psychosis symptoms) and symptom (i.e., hallucinations), which we highlighted throughout, aiming for the utmost specificity (i.e., voices/APE) as possible.

The following research questions were posed:

i. In what context do voices/APE occur, and what are these experiences like from a phenomenological perspective?
ii. Which major biopsychosocial drivers or mechanisms underlie these experiences, and how do they shape protective factors, prognostic indicators and individual outcomes?
iii. How can we consolidate and translate prevailing knowledge to develop more comprehensive aetiological models as well as effective, targeted interventions?
iv. Looking to the future, where do pertinent gaps in existing research lie, and how can these be addressed?

### Structure, methodology and quality assessment

A narrative format was elected owing to our research questions, and because existing empirical evidence was heavily skewed yet sporadic in parts. The dominance of studies in psychosis dictated these as our reference point, with further consideration of mood and anxiety disorders. Likewise, the preponderance of voices research meant this formed the foundation, with efforts to underscore the relative neglect in other sensory domains.

In terms of methodology, a structured, though not systematic search was conducted (see Fig. A in Supplementary materials). Overarching search terms (e.g., related to APE and culture) were used in conjunction with terms pertaining to each research question, with preference for systematic or meta-analytic studies (alongside recent or seminal publications) in established areas, supplemented by more comprehensive coverage involving behavioural or other empirical investigations in less established domains (e.g., other APE and diagnostic conditions). Consideration of substance-induced (and organic) hallucinations was omitted, owing to unique biological pathways implicated. Common perceptual experiences in peripheral senses (e.g., sensed presence(s), kinaesthetic-vestibular, hypnagogic-hypnopompic) were also excluded.

Lived experience involvement was actively sought throughout the stages of planning, writing and dissemination. Academic team members recruited a lived experience focus group via consumer networks in their countries of residence. Inclusion criteria were: one or more existing mental health diagnoses and/or lived experience of voices/APE, access to a smart device with internet connectivity and willingness to take part in online focus group sessions. There was no specific exclusion criterion relating to age, gender or cultural background to encourage diversity in representation. Five individuals across Australia, India and the United Kingdom, with varied ages, a range of psychiatric diagnoses, voices/APE as well as gender identities and life experiences, self-selected for involvement. Table 1 highlights pertinent lived experience themes uncovered, whereas Table A in Supplementary materials demonstrates the structure and process entailed, including the iterative process by which lived experience perspectives were incorporated. Alongside the valuable contributions, the depth of material generated in these sessions has resulted in a second publication (in preparation), specifically focused on these lived experience perspectives. Quality assessment of our narrative review based on the Scale for the Quality Assessment of Narrative Review Articles^12^ demonstrated an excellent rating of 12/12.

**Table 1:** Lived experience reflections: lessons learnt.

Topic	Theme	Elaboration
Stigma	Terminology	Appropriate, inclusive terminology: as examples, would prefer ‘hearing voices’, ‘seeing visions’ or ‘altered perceptual experiences’ The term ‘hallucination’ feels dismissive and derogatory, may have certain negative connotations (e.g., association with drug use) and does not appropriately describe voices/altered perceptual experiences
	Feeling misunderstood and misrepresented	Altered perceptual experiences and mental illness are not synonymous. More often than not, voices/altered perceptual experiences (and mental illness) are unfairly portrayed in a negative light
	Individual emotional experiences	At onset, feeling anxious, confused, lost, panicked or tense can lead to paranoia, social isolation and suicidal ideation; can be difficult to relate to people who do not have voices/altered perceptual experiences
Assessment tools	Miss core facets of lived experience	Existing tools lack the nuance to properly understand individual experiences. Most do not ask about detailed phenomenology, experiences in sensory domains outside of voices or associated life experiences
Interactions with medical professionals	Diagnosis and treatment	Being told that altered perceptual experiences are not ‘normal’ by medical staff is ostracising Initial treatments can be incredibly confronting when not warned of what to expect (e.g., rapidly transitioning from multiple complex voices to just one’s own thoughts)
	Patient involvement	Patient involvement during assessment, diagnosis, and developing treatment plans is inadequate. Often feel that symptoms or life experiences are dismissed, or that using certain words during communication with health professionals would have negative consequences. This strips individuals of their identity; feeling like ‘an uninvited guest to their own mental health’
	Intimidation	Power imbalances in medical settings need to be addressed. Cultural and socioeconomic differences may create stress and can be triggering. The existence and effects of this are largely unconsidered in research and clinical practice
Contributing factors	Life history	Experiences during adolescence: depression and other symptoms often start in adolescence Negative life events: trauma, negative memories, domestic violence
	Current mental state	Mood fluctuations, anxiety and irritability, stress levels, lack of sleep or insomnia, social isolation or feeling lonely
Treatments	Current treatments	Psychological therapies: not ‘one-size-fits-all’; those targeted at individual skillsets and goals for quality of life improvements work best Brain stimulation: mixed results, helpful for some persons. Discussions with trusted medical staff, and making informed decisions are key Pharmacology: Side effects need to be addressed
	Important considerations	More sensitive use of language, and patient involvement in own treatment. Holistic treatments which focus on sleep and negative life events (e.g., trauma). Focusing on multimodal and multisensory voices/altered perceptual experiences
Transcultural considerations	Underrepresented aspects of culture and subculture	Gender: cisgender women and transgender individuals Race: non-Caucasian representation Life experiences: including individuals with experiences of domestic violence, coercive relationships or sex work
	Family dynamics	Intergenerational communication gaps Dual cultural heritage: differing stance on mental health, and how to communicate between cultures (e.g., some cultures believe Altered perceptual experiences are akin to possession; this may impact an individual’s ability to relate to family members)
Future directions	Technological advances	Use of artificial intelligence in research and practice will inevitably create bias, and should be managed carefully
	Professional training	Lived experience involvement should be incorporated in the training of medical staff and students, particularly those with significant patient-facing roles (e.g., nurses, paramedics)

### Transcultural considerations

Culture may be defined as comprising a shared set of ‘knowledge, belief, art, law, morals, custom and any other capabilities and habits acquired’ for adaptive function within one’s social milieu^13^. This construct operates across multiple intersections (e.g., ethnicity/race, nationality and religion), but can encompass subcultural perspectives derived from membership in other social groups. Established knowledge on hallucinations mostly rests on the assumptions of a preconceived ‘Western biomedical model’, with relative neglect of other cultural viewpoints. Yet culture exerts a notable impact on voices/APE in terms of: (i) their definition (i.e., what constitutes a hallucination) and thus prevalence; (ii) significance personally and within one’s broader worldview (e.g., meaning-making endeavours); and (iii) how they are managed (e.g., extent of pathologisation) and ultimately, individual outcomes^14,15^. Hitherto neglected but critical transcultural considerations were embedded throughout where possible, with culture as an experimental condition examined explicitly in available studies.

## Phenomenological characterisation of voices/APE

### Assessment tools

Table 2 describes common standardised assessment tools identified by the review. These instruments evaluate varied aspects of phenomenology, including perceptual (e.g., frequency), cognitive (e.g., insight), and emotional (e.g., distress) characteristics, and are typically employed for screening purposes in clinical settings as well as drug trials and mechanistic studies. Several well-validated measures characterising the nature and severity of voices in psychosis are widely used, e.g., ref. ^16^. Other tools have looked beyond the auditory domain, but have not shown equal consideration for all sensory modalities, e.g., ref. ^17^, or only elicit limited detail, e.g., ref. ^18^. Some comprise a component of broader psychosis measures, where evaluation of APE may be based primarily on voices (and/or reduced to a single hallucination score) e.g., ref. ^19^. Many visual hallucinations measures stem from the neurological field, resulting in associated drawbacks accounting for specific illness effects (e.g., Parkinson’s disease), although transdiagnostic tools have started to emerge, e.g., ref. ^20^. Existing instruments have thus been largely validated in psychosis (or neurological) populations, but not cross-validated for use in cohorts predominantly experiencing mood or anxiety disorders. Critiques of existing measures have also been offered^21–23^. To our knowledge, validated assessments evaluating unimodal somatic-tactile, olfactory, or gustatory hallucinations have yet to be developed. Instruments rating unimodal or multisensory, e.g., ref. ^24^ experiences in the general population do exist, and are important for establishing parallels (and highlighting contrasts), given voices/APE transpire as a variation of typical human experience.

**Table 2:** Widely used assessment tools, including associated strengths and limitations.

Name of measure, citation	Modalities investigated	Format, # of questions (# of APE questions)	Validated population(s)	Validated languages	Variants	Phenomenological facet(s) assessed	Strengths	Limitations	# of citations^+^
Scale for the Assessment of Positive Symptoms SAPS^18^;	AH VH S/TH OH	Semi-structured, researcher-rated, 6-point Likert scale 35 (10)	SSD PD^229,230^	English Chinese^229^ Spanish	Short SAPS for PD^230^ Enhanced SAPS for PD^231^	Content: AVH commenting, conversing; overall severity	High citations enable comparison across studies; many sensory modalities covered	Created to assess wider SSD symptoms, thus not comprehensive; single question per sensory modality	5,029
Cardiff Anomalous Perceptions Scale CAPS^232^;	AH VH S/TH OH GH Other^§^	Structured, self-report, 5-point Likert scale 32 (32)	SSD BD MDD^4^ MDD-P GAX^233^ NC Youth^234^	English Spanish^235^ Gujarati^236^ Hokkien^237^	-	Distress; frequency; intrusiveness	Validated in a wide variety of demographics; robust brief overview of phenomenology	Does not assess detailed phenomenological information	320
Mental Health Research Institute Unusual Perceptions Schedule MUPS^238^;	AH VH S/TH OH OH Other^§^	Semi-structured, researcher-rated, binary, multiple-point Likert scales 70 (39)	SSD	English	-	Content (1^st^/2^nd^/3^rd^ person, affect, conversing, commenting, gender, social and subverbal qualities); insight; location; timing (diurnal variation, duration, frequency)	Comprehensive AVH assessment. Quantitative and qualitative sections	Long administration time (~45 min); predominant focus on AH	201
Parkinson’s Disease-Associated Psychotic Symptoms Questionnaire RSPS^239^;	VH Other^§^	Structured, self-report, binary 10 (4)	PD	English	-	Content	Brief administration time (~10 min)	Does not assess detailed phenomenological information	122
Psychotic Symptoms Rating Scale PSYRATS^16^;	AH	Structured, researcher-rated, 5-point Likert scale 18 (12)	SSD Intellectual disability^240^	English Chinese^241^ French^242^ Indonesian^243^ Portuguese^244^ Malay^245^ Turkish^246^	-	Beliefs of origin; content (affect, loudness); control; disruption; distress; location; timing (duration, frequency)	Validated in a wide variety of demographics; brief administration time	Does not assess detailed phenomenological information	1,626
Positive and Negative Syndrome Scale PANSS^19^;	General	Semi-structured, researcher-rated, 7-point scale 40 (1)	SSD	English Arabic^247^ Chinese^248^ Russian^249^	-	Hallucinations (not modality-specific)	High citations enables comparison across studies	Relatively long administration time for one hallucination item, not modality-specific	22,697
Launay Slade Hallucination Scale LSHS^250,251^;	AH VH	Structured, self-report, 5-point Likert scale 13 (4)	SSD NC	English Hindi^252^ Korean^253^	Revised^254^ 2-factor structure^255^ 3-factor structure^256^ Extended multimodal^257^	Content; distress	Brief administration time	Numerous versions preclude comparisons across studies and reduce consistency	641
North East Visual Hallucinations Interview NEVHI^258^;	VH Other^§^	Semi-structured, researcher-rated, binary, open-ended, 3-point Likert scale 20 (20)	Older adults DLB Eye disease PD^259^	English	-	Behavioural interaction; beliefs of origin; content (complexity, emotion); control; timing (diurnal variation, duration, frequency, situational occurrence)	Quantitative and qualitative sections; multiple timeframes assessed (past month, lifetime); Comprehensive for past month only	Relatively long administration time	131
Questionnaire for Psychotic Experiences QPE^17^;	AH VH S/TH OH Other^§^	Semi-structured, researcher-rated, multiple point Likert scales 50 (33)	SSD	English Arabic^231^	-	Behavioural interaction; content (AH, VH: command, complexity, emotion, repetition); distress; impact; insight; location; timing (diurnal variation, duration, frequency)	Comprehensive for AH/VH only; multiple timeframes assessed (past 7 days, lifetime)	Relatively long administration time; no GH questions	64

The measures included in this table are not exhaustive, and merely aim to provide an illustration of the range of assessment tools available. Measures have been ordered alphabetically, as per first author. Validated languages and variants may not be exhaustive, original authors should be contacted directly. Only English literature was searched. Citations have been included to contextualise how widely each measure has been used. +: As of July 2023; §: Other may include: dreams, coping strategies, delusions associated with PE, hypnagogic-hypnopompic experiences, intrusive thoughts, sensed presences, temporal lobe function, vivid daydreams; *APE* Altered perceptual experiences, *Modalities:* *AH* Auditory hallucination, *GH* Gustatory hallucination, *OH* Olfactory hallucination, *S/TH* Somatic-tactile hallucination, *VH* Visual hallucination; *Populations:* BD: Bipolar disorder; *BPD*: Borderline personality disorder; *GAX*: Generalised anxiety disorder; *LBD*: Dementia with Lewy Bodies; *NC*: Non-clinical; *MDD*: Major depressive disorder; *MDD-P*: MDD with psychotic symptoms; *PD*: Parkinson’s disease; *SSD*: Schizophrenia spectrum disorders.

### Prevalence of hallucinations across the sensory modalities

Table 3 provides an overview of the prevalence of unimodal and multisensory/multimodal hallucinations across several psychiatric and neurological populations. Wide-ranging estimates were noted, depending on the sensory modality and diagnostic condition under consideration, also owing to differing operationalisations and tools employed across studies. Empirical data for certain sensory modalities (e.g., gustatory) was notably missing. These varying and sporadic figures, accompanied by assessment limitations, render it difficult to discern consistent patterns regarding prevalence.

**Table 3:** Prevalence of multisensory hallucinations across a range of population groups.

Diagnosis	Type of paper^a^	Modality^b^	Prevalence
**Psychiatric conditions**			
Schizophrenia spectrum disorders	Large-scale empirical^5^ Narrative review^6^ Large-scale empirical^5^	AH VH S/TH OH GH Multisensory	64–80% 23–31% 9–19% 6–10% 1–31% 30–97%
Bipolar disorder	Systematic review^71^ Large-scale empirical^87^	AH VH S/TH OH/GH	11–63% 26% 29% 17%
Major depressive disorder	Systematic review^71^ Large-scale empirical^87^	AH VH S/TH OH, GH	5–40% 23% 39% 12%
Anxiety disorders^§^ Generalised anxiety disorder	Large-scale empirical^260^ Large-scale empirical^35^	AH VH S/TH OH GH General	9% 5% 3% 2% 2% 5–17%
Obsessive-compulsive disorder	Small-scale empirical^77^ Case series^76^	AH VH S/TH OH GH	37% n.s. n.s. n.s. n.s.
Borderline personality disorder	Systematic review^261^ Epidemiological^82^	AH VH S/TH OH GH Multisensory	22–50% 11–50% 15–47% 17–31% 8% 50%
Dissociative identity disorder	Epidemiological^79^	AH VH S/TH OH GH	55–83% 83% 90% 76% 55%
Post-traumatic stress disorder	Small-scale empirical^262^	AH	50%
**Neurological conditions**			
Alzheimer’s disease	Narrative review^263^	AH VH S/TH, OH, GH	1–29% 4–59% 0.4–9%
Epilepsy	Narrative review^27^ Narrative review^38^	AH VH S/TH OH GH	14% 8–72% n.s. 5% 4%
Eye disease	Narrative review^264^ Narrative review^217^ Narrative review^6^	AH VH Multisensory	n.s. 15–60%^~^ 4%
Lewy body disease	Meta-analysis^42^ Narrative review^6^	AH VH Multisensory	31% 62% 32%
Migraine/headache disorders	Case series^265^ Systematic review^40^ Small-scale empirical^266^	AH VH OH	0.2% n.s. 1%
Narcolepsy	Systematic review^41^ Small-scale empirical^267^ Systematic review^41^	AH VH OH S/TH Multisensory	86% 40% 28% 48% 38%
Parkinson’s disease	Meta-analysis^42^ Small-scale empirical^268^ Small-scale empirical^269^ Narrative review^6^	AH VH S/TH OH Multisensory	9% 28% 20% 10% 10%
**General population**			
Non-clinical	Narrative review^27^ Small-scale empirical^270^ Epidemiological^271^	AH VH S/TH OH Multisensory/ multimodal	10–15% 17% 7% 11% 1–12%
Grief/ bereavement	Narrative review^155^	AH VH	13–50% 14–79%

a: *N* ≥ 500 for large-scale empirical study, else denoted as small-scale empirical study; b: Missing information on the prevalence of modalities in some diagnoses is due to a lack of empirical data; c: Anxiety disorders have been included as a whole, due to a lack of information on specific anxiety disorders. Anxiety disorders in this study included generalised anxiety disorder, panic disorder and social anxiety; ~: Depending on the degree of visual loss; n.s.: Presence reported, but prevalence as yet unknown; *Modalities:* *AH* Auditory hallucination, *GH* Gustatory hallucination, *OH* Olfactory hallucination, *S/TH* Somatic-tactile hallucination, *VH* Visual hallucination.

### Phenomenological characterisation in psychosis

An early empirical study to phenomenologically quantify voices in psychosis^25^ has been largely supported by subsequent works homing in on particular characteristics, for instance, personification^26^. Several comprehensive reviews have also summarised phenomenological studies to date^27–30^. Broadly speaking, hearing voices is a fundamentally heterogeneous experience that fluctuates not just from one individual to the next, but also likely within the same person over time. Yet it is common for voices to be experienced with similar clarity and volume as everyday conversations, and involve speaking to or about the hearer. Few characteristics of voices appear specific to psychosis, although greater frequency, negative content (involving critiques, threats or commands), and distress may be reported in schizophrenia^31^. Emotional distress seems to be the primary facet differentiating clinical voices from similar experiences reported in the general population^27,28,32–35^. It is thus critical to recognise that hallucinations exist on a continuum ranging from mild, transient events to persistent, distressing experiences necessitating psychiatric care, accompanied by substantial variation across experiential facets^29,31,36^.

The study of visual (and olfactory) hallucinations has primarily advanced within neurology, including Alzheimer’s disease^37^, epilepsy^38^, eye disease^39^, migraine^40^, narcolepsy^41^ and Parkinson’s and Lewy body diseases^42^. Their relative neglect in psychiatry is being remedied by increasing attention of late^43–46^; the presence of visual hallucinations has tentatively been linked to negative imagery, greater illness severity and less favourable outcomes^39,44^. An even more inclusive shift has recently transpired, involving their study in conjunction with auditory^7,47,48^ or non-auditory^49,50^ domains, or within a broader multisensory framework^4–6,51^. Case reports abound e.g., ref. ^52^, including an early account of “fused” multimodal hallucinations as “seeing voices”^53^. Multisensory/multimodal^4^ hallucinations seem more typical in psychosis than unimodal events, with the presence of visual (but not auditory) modality conferring increased risk of hallucinations in other senses^5^, and multisensory involvement triggering heightened conviction, delusional ideation and distress^48–51^. Although limited, studies into unimodal somatic-tactile, olfactory and gustatory hallucinations suggest a higher incidence than previously held^54–57^, and have also identified associations with delusionality and depressive episodes^54,55^.

#### Consideration of broader, multidisciplinary perspectives, including qualitative studies

There has been a move towards incorporating sociocultural, historical, and experiential viewpoints via diverse methodologies (e.g., video diaries, body maps)^58,59^, and fusing psychiatric/psychological theories with anthropological, ethnographic, or philosophical insights^15^. A proliferation of rich, qualitative studies has ensued, also incorporating lived experience perspectives^26,60–66^. Notable observations include: (i) experiences of “thought-like” or “soundless” voices^66^; (ii) hearers forming embodied relationships with characterological identities^26,60,63,65,66^; (iii) involvement of multiple bodily sensations^26,65,66^; (iv) associations with positive or neutral emotions^66^; and (v) a focus on acceptance, meaning-making, and recovery^60–62,64^. Contributions have stemmed from a few studies, including persons with other diagnostic conditions^64,66–68^, but advancements have been mainly limited to voice-hearing, with one known study examining multimodal hallucinations^69^.

### Phenomenological characterisation in mood and anxiety

Limited literature has characterised voices/APE in bipolar and depressive disorders. Two systematic reviews concluded that voices remain a major but largely unstudied symptom, with a distinct neglect of fluctuating mood states prominent in these disorders^70,71^. Recent studies comparing voice-hearing in mood disorders with schizophrenia spectrum disorders noted more phenomenological similarities than differences^72–74^. There were however, minor exceptions, where voice-related distress was predicted by disparate factors (e.g., voice resistance) in bipolar disorder, linked to discrepancies in cognitive appraisals that may convey therapeutic implications.

**Fig. 1:**
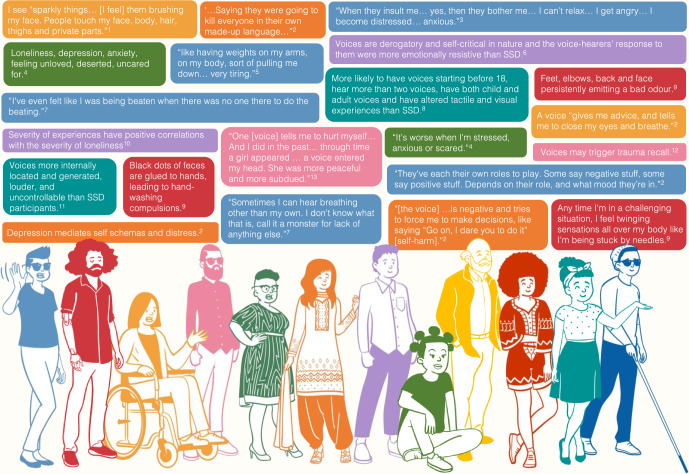
Sample phenomenological descriptors of voices/APE in conditions associated with psychosis, mood and anxiety. Quotes were selected from highly cited qualitative research in the field and aimed to give a broad overview of the subjective experiences of voices/APE. SSD: schizophrenia spectrum disorders. Each colour represents a different condition associated with psychosis, mood and anxiety, as follows: blue: SSD; green: Generalised anxiety disorder; orange: Major depressive disorder; pink: Post-traumatic stress disorder; purple: Borderline personality disorder; red: Obsessive-compulsive disorder; teal: Dissociative identity disorder; yellow: Bipolar disorder. 1^299^; 2^300^; 3^301^; 4^302^; 5^69^; 6^81^; 7^303^; 8^79^; 9^76^; 10^83^; 11^78^; 12^93^; 13^67^.

There has also been scant research into voices/APE in anxiety conditions, with a single case report documenting olfactory hallucinations in generalised anxiety disorder^75^. Sporadic accounts exist for obsessive-compulsive disorder^76,77^, dissociative identity disorder^78,79^, borderline personality disorder^68,80–83^ and post-traumatic stress disorder^46,67,68,82,84^. Interpretation is complicated by pathognomonic symptoms within these disorders that can be difficult to distinguish from hallucinations, raising further definitional issues^84^. A lack of comparative studies precludes firm conclusions regarding phenomenological differences (or similarities) relative to psychotic and mood disorders. Yet a striking observation is rich descriptions of multisensory hallucinations in these latter conditions^46,76,79,81,82^, such as voices in the form of “loud, sensory-laden” thoughts in obsessive-compulsive disorder^77^. Figure 1 shows sample descriptors characterising voices/APE across the diagnostic conditions considered, illustrating the wide diversity in these experiences.

Given disorder comorbidities, symptom overlaps, and the limited diagnostic utility of hallucination phenomenology^10^, it may be prudent to examine voices/APE from the stance of dimensional mood and anxiety. To this end, depression^85,86^ and anxiety^85,87,88^ correlated with the presence/severity, negative content, and distress elicited by voices across these psychiatric conditions. Yet associations between the presence of voices/APE with symptoms of depression and anxiety are likely multifarious, with intricate links to other constructs. Content-wise, for example, commands to self-harm have been predicted by depression and anxiety, with critical and threatening voices predicted by anxiety^89^. Depression and anxiety^90^ moreover, exert mutual influences, with depression also known to mediate or moderate relationships between voices and negative core schemas^91^ or suicidal ideation^92^ respectively.

### Transcultural research

Transcultural research into voices/APE has touched on diverse geographical regions, such as Cambodia^93^, Egypt^94^, Ghana^95^, India^96^ or Indonesia^97^, and in relation to religious beliefs^98,99^. However, few studies have explicitly considered transcultural perspectives. Limited comparative studies have sought to highlight differences (and similarities) in voice-hearing across the USA (e.g., featuring diagnostic labels and violent voices), Ghana (e.g., morally superior and omnipotent voices), and India (e.g., cultivating relationships with voices that provide guidance), illustrating how powerful cultural beliefs can be in shaping experiential facets^15,95^. Heightened distress has been linked to a “ghost summoning”^93^ or jinn encounter^98,99^, showing that explanatory models are partly dictated by cultural attitudes. Cultural factors may also promote a normalising view, where living with voices is seen as part of everyday life^97^. There has also been growing interest in how spirituality and voices/APE may exert mutual influences, with implications for therapeutic innovation. For instance, voices/APE experienced by mediums or spiritualists are embraced, with deliberate practice devoted to the cultivation of such altered perceptual states^100–102^.

Another impactful, yet often neglected, subcultural perspective relates to gender (also see Table 1 for lived experience perspectives). Power imbalances inherent in extant social structures influence how voices/APE are perceived and responded to, whether in clinical settings or broader social situations. A gendered account of female voice-hearing has noted the role of trauma (see section on ‘Role of trauma’) as well as parallels between women’s relationships with their voices and persons in their social world^103^. Yet there have been minimal efforts to replicate or extend such research, including consideration of non-binary and transgender perspectives.

The international Hearing Voices Movement (HVM) is a service-user co-led group espousing voice-hearing as a meaningful human experience, and promoting the priorities and viewpoints of lived experience experts^8^. A recent systematic review (comprising mainly qualitative research) of peer support groups based on HVM principles concluded potential benefits to members including socialisation, meaning-making, and promoting hope for recovery^104^.

## Biopsychosocial mechanisms underlying voices/APE

### Role of trauma

Many psychotic, mood and anxiety conditions have demonstrated complex links with personal trauma history^105–107^, perhaps explicitly so in borderline personality disorder^108^, dissociative identity disorder^79^, and post-traumatic stress disorder^84^. It is therefore unsurprising that voices/APE (and attendant psychiatric diagnoses) predominate in adolescence^109^, where major life traumas can arise^110^. Trauma is increasingly accepted as a significant contributor to psychotic experiences, a shift from earlier conceptions, with some scholars now proposing a unified traumagenic neurodevelopmental model of psychosis^111,112^. Yet a key limitation lies in its lack of specificity, regarding symptom differentiation and the nature of traumatic exposures. As an illustration, trauma associated with an intention to harm has been specifically related to the emergence of hallucinations^113^, with conspicuous links between childhood sexual abuse and voices^114^. Tentative associations between trauma and hallucination attribution^115^, content^114,116^, and severity^117^ have been noted, with discrete phenomenological patterns likely reflecting differing trauma salience^118^. Underlying mediators involving dissociative processes, emotional dysregulation and negative schemas have likewise been put forward^119–121^.

There is growing acknowledgement that a significant trauma history is also associated with increased endorsement of visual hallucinations^43,46,122,123^. Prevailing findings applicable to voices may thus selectively extend to other sensory domains e.g., ref. ^124^. A dose-response relationship has been suggested between cumulative trauma and number of sensory modalities involved^125,126^, evident even in child cohorts^127^. Emerging qualitative and transcultural perspectives^128,129^ have likewise facilitated an inclusive understanding of diverse, nuanced impacts of trauma on voices/APE.

### Neurocognition

Another area of prolific research into voices (and non-verbal sounds) relates to neurocognitive correlates. Full consideration of this extensive literature is beyond the present scope. However, authoritative reviews pertaining to the domains of executive function and inhibition^130^, linguistic processing^131^, neuroanatomical correlates^132^ and resting state connectivity^133^, prediction errors/overdominance of priors^134^, as well as self- or source-monitoring^135^ exist (also see Table B pertaining to aetiological models). A paradox emerges, in that an apparent lack, yet also an over-specificity of investigations relating to voices. This is exemplified by emphasis on a broad disorder level (e.g., schizophrenia), alongside neglect of nuanced consideration regarding how these mechanisms may operate for non-auditory hallucinations. There is also a question of whether neurocognitive mechanisms are truly aetiological or perhaps stem from other illness effects, which merely perpetuate voices following onset.

Studies into neurocognitive mechanisms associated with non-auditory hallucinations in psychosis (or other psychiatric conditions) remain scant. Aberrant source-monitoring^136,137^, visual processing^138^, and hippocampal connectivity^139^ or conversely intact attentional^140^ and visual recognition^141^ performance have been tentatively documented in relation to visual hallucinations. Preliminary evidence of socioemotional/decision-making deficits related to orbitofrontal cortex function^142^ as well as modality-specific source-monitoring difficulties^143^ in olfactory hallucinations have likewise been recorded. Psychiatric research comparing multisensory processing^144^ and general cognitive function linked to multisensory/multimodal hallucinations appears lacking. An inclusive viewpoint, encompassing how existing neurocognitive processes may be selectively applied to aid mechanistic explanations of multisensory hallucinations (i.e., modality-general versus modality-specific) has been proposed^145^, and warrants conceptual and empirical corroboration.

### Other biopsychosocial drivers

A host of other biopsychosocial drivers are known to contribute to voices/APE. A genetic basis for voices in psychosis has been proposed^146^, with *dysbindin* variants linked to auditory, visual, and olfactory hallucinations in a modality-specific manner^147^. Increased striatal dopamine^148^ or inter-regional excitatory/inhibitory imbalances^149^ offer credible neurotransmitter dysregulation accounts for voices, while identification of shared familial history of diagnostic conditions (although inconclusive) points towards possible heritability effects^10,150^. Personality factors, involving absorption^102,151–154^ or schizotypy^27,83,150^, may directly shape voices/APE, albeit in culturally distinct and meaningful ways^100,152^. Environmental determinants, ranging from grief/bereavement^155^ to sensory^156^ or sleep^157^ deprivation, and social withdrawal^158,159^ exert myriad influences. These elements operate collectively across individuals within their broader sociocultural milieu to ultimately determine how voices/APE are perceived and managed. Table 4 summarises existing empirical support (or lack thereof) for these biopsychosocial mechanisms, illustrating how mechanistic research involving the non-auditory modalities is still largely lacking.

**Table 4:** Summary of empirical support (or lack thereof) for biopsychosocial mechanisms underlying voices/APE.

Biopsychosocial mechanism	AH	VH	OH	S/TH	GH
**Environmental and social determinants**					
Grief/bereavement^155^	✓	✓	✓	✓	-
Sensory deprivation^156^	✓	✓	-	✓	-
Sleep deprivation^157^	✓	✓	-	✓	-
Social deafferentation^158,159^	✓	×	-	✓	-
**Genetics and heritability**					
Dysbindin^147^	✓	✓	✓	-	-
Familial history of psychiatric illness^10,55,150^	?	?	?	?	?
Inter-regional excitatory/inhibitory balance^149,272,273^	✓	✓	✓	-	✓
Striatal dopamine^148^	✓	-	-	-	-
**Neurocognition**					
Executive function and inhibition^130,142^	✓	✓	✓	-	-
Neuroanatomical correlates^132,190,274^	✓	✓	✓	✓	-
Predictive coding^134^	✓	✓	-	-	-
Resting state connectivity^133,139,275,276^	✓	✓	✓	-	-
Self/source-monitoring^135–137,143^	?	✓	✓	-	-
Sensory processing^131,138^	✓	✓	-	-	-
**Personality**					
Absorption^102,151–154^	✓	?	✓	×	✓
Depersonalisation^124^	✓	?	✓	?	×
Schizotypy^27,83^	✓	✓	✓	✓	-
**Trauma**					
Abuse: physical, sexual, emotional ^125,126^	✓	✓	✓	✓	✓
Neglect with intention to harm^113^	✓	✓	✓	✓	-
Neglect without intention to harm^113^	×	-	-	-	-

Extant evidence of biopsychosocial mechanisms underlying multisensory/multimodal APE does not allow for their meaningful inclusion in table. ✓: Evidence; *×*: No evidence;?: Contrary evidence exists, otherwise some support in the form of case studies; -: No known studies in the area. *AH* Auditory hallucination, *GH* Gustatory hallucination, *OH* Olfactory hallucination, *S/TH* Somatictactile hallucination, *VH* Visual hallucination.

### Resilience, prognosis and outcomes

Beyond risk, it is imperative to consider resilience in mitigating prognoses and outcomes related to distressing voices/APE^160^. Limited literature has pointed to the protective influence of age of onset^10,31,56^ and religion^161^ for voices in schizophrenia. Outside of adolescence^162^, voices/APE in childhood are often multisensory but transitory^109^, with middle and old age similarly conferring protection^35,163^. Religion is also known to offer contextualised significance for voices/APE in clinical and non-clinical populations^161^, confounded by cultural beliefs and practices^164,165^. Preliminary evidence has suggested that transition to psychosis may be exacerbated by mood difficulties^31^, with emotional stability bestowing protection^56^. This observation may be pertinent for bipolar and depressive disorders, where mood fluctuations are prominent^72,73^. Whether these protective effects generalise to other psychiatric conditions remains unknown.

Conclusions regarding prognoses and long-term outcomes are best derived from longitudinal studies, though specificity issues are once more a limitation, given that reporting of voices/APE per se (as opposed to syndromes or disorders) is scant. Some research has focused on children/adolescents, e.g., refs. ^166,167^, whereas existing adult studies tend to be small-scale and/or lack long-term follow-up, e.g., refs. ^61,168^. Two robust 20-year studies form the exception^169,170^. Tracking the longitudinal course of hallucinations informed differential diagnosis of schizophrenia versus bipolar disorder, with early and frequent voices/APE predicting low recovery and occupational function respectively^169^. Presence of voices conversing/commenting was noted in both schizophrenia and bipolar disorder, but more disabling and related to less favourable long-term outcomes in the former^170^. Another emerging prognostic indicator pertains to multisensory hallucinations, where comorbid involvement of the visual modality has been tentatively linked to more severe psychopathology, greater disability and relapse, and poorer functional outcomes^39,44,171^.

## Aetiological models and interventions

### Subtyping of voices/APE

Given the heterogeneity in voices/APE, nascent efforts at identifying voice phenotypes^44^ may potentially contribute to aetiological and therapeutic advances^172,173^. A previous robust attempt yielded four voice subtypes—Constant commanding and commenting, Replay, Own thoughts, and Non-verbal^174^, although subtype overlaps within participants underscore inherent clinical complexities. Other studies have attempted to cluster also by phenomenology^175^, trauma^176^ or symptom comorbidity^177^. There has been a lack of specificity (i.e., disorder-specific) with regards to subtyping by cognition, e.g., ref. ^178^, and clustering in visual^171^ and olfactory^57^ domains is still in its infancy. When broader multisensory and diagnostic considerations are taken in account, it remains to be seen whether a subtyping approach would yield further clinical and research utility.

### Aetiological models for voices/APE

Endeavours have been made to develop neurocognitive mechanisms into plausible aetiological models. Although a comprehensive overview of aetiological theories is beyond the present scope, Table B in Supplementary materials provides a summary of prevailing models, including critiques asking how existing theories may be applied to other sensory modalities. Current theories evidently stem from consideration of voices in psychosis. These include the inhibition/memory^179,180^ or inner speech models^181,182^, and more recently, the excitatory/inhibitory imbalance hypothesis^183^ or interhemispheric miscommunication theory^184^. Review and critique of neurocognitive models has revealed mixed empirical and theoretical support, e.g., ref. ^30,185,186^, with an inability to account for the diversity of these experiences proving to be a significant drawback^187^. A phenomenologically specific theory of how negative voice content is derived across biopsychosocial levels, incorporating cultural influences, has been proposed^188^, with supporting evidence transdiagnostically and transculturally^89^ though contrary evidence exists. Although aetiology has also seen some progress in relation to trauma, including biopsychosocial and neurobiological evidence, these models tend to operate on a syndrome or disorder level^111,112^.

Given the relative lack of visual hallucination models in psychiatry^39^, credible theories may be borrowed from the neurological sphere (also see Table B). Emerging interdisciplinary collaborations suggest the potential for fruitful consolidation of prevailing aetiological knowledge regarding auditory and visual hallucinations^189^, although somatic-tactile, olfactory and gustatory modalities remain comparatively neglected. There have also been calls to revise and integrate existing unimodal causative theories to account for multisensory/multimodal hallucinations^6^. Mechanistic studies beyond the auditory domain, e.g., refs. ^132,190^, applied to disorders other than psychosis, e.g., refs. ^132,191^, and calling for explicit consideration of culture^186^, are emerging. However, ultimately, a major paradigm shift is needed, where inclusive explanatory models encompassing experiential elements of voices/APE relating to diverse interactions amongst noted biopsychosocial mechanisms are developed.

### Specific interventions for voices/APE

Despite a considerable treatment literature, specificity again is an issue. Interventions rarely target voices/APE, but rather apply generally on a syndrome (e.g., psychosis) or disorder (e.g., schizophrenia) level, e.g., refs. ^192,193^. Importantly, voice-specific interventions do not routinely consider the characteristics of individual experiences, and treatments (employing randomised-controlled trials; RCTs) directed at distressing experiences in other sensory domains within psychiatry, e.g., refs. ^194,195^ and across non-psychotic diagnostic conditions, e.g., ref.^196^ remain inadequate.

Broadly speaking, voice-specific interventions can be divided into pharmacological^2,196^, neurostimulation^197,198^, and psychological^199–201^ avenues. An inclusive review of existing interventions is beyond the present scope, but Table 5 provides a summary of prevailing therapeutic options, including available empirical support and limitations. Robust efficacy was noted for antipsychotic medication, including their broader value in reducing psychotic symptoms within disorders. There is general consensus that no atypical antipsychotic demonstrated superiority over others in treating voices/APE, but side effect profiles may differ markedly by drug and patient. Similarly, neurostimulation outcomes have also shown varying evidence of benefits, which appear dependent on the cortical region(s) targeted.

**Table 5:** Prevailing treatments targeted at voices, including available empirical support and limitations.

Treatment	Summary	Empirical support	Type of paper	Limitations
**Pharmacology**				
Antipsychotic medication	Includes first (e.g., chlorpromazine, haloperidol) and second (e.g., clozapine, olanzapine) generation antipsychotic medications and adjunct medications targeting mood symptoms (e.g., benzodiazepine)	Meta-analyses show an established effect on positive symptoms of psychosis, but analysis of evidence of efficacy for hallucinations specifically is limited. No evidence of any antipsychotic demonstrates superior efficacy over others for AH^2,196^	Narrative review^2^, systematic review^196^	Individual variation in treatment response. Numerous side effects, depending on specific medication, including excessive saliva (78% of individuals), altered appetite (63%), sedation (44%), weight gain (44%), dry mouth (38%), nausea (29%), dizziness (22%), irritability (18%), akathisia (15%), insomnia (14%), confusion (13%), tremors (11%), anxiety (11%) and headaches 11%^196^
**Neurostimulation**				
Electroconvulsive therapy	Application of a unidirectional electrical current to the scalp to induce generalised seizure. Performed under general anaesthesia and in conjunction with muscle relaxants	No known effects on AH severity, evidence of reduced depression^2^	Review of meta analyses^2^	Although more generalised clinical improvements have been shown in SSD populations, a specific reduction in hallucination severity has never been demonstrated^2^
Transcranial direct current stimulation	Application of a painless low unidirectional electrical current to the scalp to elicit changes in cortical activity^277^	Contrary evidence^278,279^; support for reduced AH severity when targeting the frontoparietal network^278^	Meta-analysis^278^; systematic review^279^	Underlying mechanism of action still unknown
Repetitive transcranial magnetic stimulation	Application of painless high-intensity magnetic pulses to the scalp to induce electrical activity in the underlying cortical region over several days^198^	Contrary evidence^279,280^; support for reduced AH severity, particularly with a 1 Hz pulse applied over the left temporoparietal area^280^	Systematic review^279^; meta-analysis^280^	Effects in unmedicated individuals unknown^198^; efficacy may be reliant on individual cortical vasculature^197^
Real-time neurofeedback	Utilises functional magnetic resonance imaging in conjunction with a visual feedback interface to train individuals to upregulate activity of specific cortical regions^197^	No meta-analyses conducted; evidence of reduced AH severity, and beliefs of voice origin, associated with increased anterior cingulate and decreased left superior temporal gyrus activity^197,281^	Narrative review^281^; pilot study^197^	Reliance on expensive technology; underlying mechanism of action still unknown
**Psychological**				
Acceptance and commitment therapy (ACT)	Assists individuals to disengage attention from, and reduce habitual behavioural responses to, salient aspects of voices i.e., intrusiveness, negative content and interpersonal qualities^282^	Reduced anxiety, depression, rehospitalisation rates and voice-related distress and improved quality of life^283^	Review of meta analyses^283^	Effects on persisting AH yet to be established^192^
Cognitive behavioural therapy for psychosis (CBTp)	Collaborative approach aimed at reframing appraisals and modifying behaviour, with an overarching aim of reducing distress, improving functioning and wellbeing^199^. Provides the basis of many psychological therapeutic approaches	Decreased AH severity in trials of formulation-based CBTp. In trials focusing on AH-specific protocols,^284^ long-term decreases in perceived power of commanding AH^285^, some evidence of decreased AH severity, distress and depression^199^	Meta-analysis^284^; narrative review^199,285^	Many randomised control trials of focused interventions for voices are underpowered, limiting conclusions^199^. Structured approach may be challenging for some
Creative arts therapies	Adjunct therapies using creative means (e.g., art, music) to facilitate verbal and non-verbal appraisals of mental health^286^	Reduced AH severity, anxiety and depression, improved social functioning and quality of life^287,288^	Meta-analysis^287,288^	Limited research in AH
Mindfulness-based approaches	Cultivates a daily meditation practice to experience voices and other mental phenomena without judgement^289^	Highly efficacious reductions in depression, and reductions in overall measures of psychosis symptoms^192^	Pilot study^192^	No specific effect on AH severity or distress^192^
Relational therapies	Use experiential role-playing to establish or re-evaluate the relationship between voice(s) and voice-hearer^290^, include Relating Therapy, Talking with Voices and Avatar Therapy (which uses digital representations of voices to aid therapy)	Reduced AH severity and distress, decreased anxiety and depression, and increased self-esteem^209^	Systematic review^209^	Role-playing may be challenging and distressing for some individuals
Smartphone applications	Numerous versions e.g., refs. ^202,212^ which apply ecological momentary interventions (e.g., coping strategies)	No meta-analyses conducted; evidence of decreased AH severity, reduced distress, and improved coping^291^	Case series^291^	Time involvement can be onerous; requires individuals to have access to and be comfortable using a smartphone
Trauma-informed therapies	Numerous therapies (e.g., exposure-based, eye movement desation and reprocessing (EMDR)) aimed at managing post-traumatic stress disorder symptoms^292^	Contrary evidence, which appears to vary depending on construct being tested^210,211,292,293^; evidence of reduced AH severity, decreased distress and perceived recovery when dissociation or intrusive thoughts are targeted^211,293^	Case series^293^; pilot study^210^; narrative review^292^; meta-analysis^211^	Possibly more efficacious for ameliorating delusional symptoms than AH^292,293^
Self-management programmes	Self-help books and self-directed computer-based programmes to promote self-management of AH ^294–296^	When supported by a practitioner, self-management appears feasible, and pilot trial results suggest decreases in AH severity	Pilot studies^294–296^	Awaiting results of full randomised controlled trials. Self-guided format may be challenging for some, and literacy and digital literacy may be barriers
Hearing voices groups	Peer support groups aiming to promote sharing of experiences and coping strategies	A range of benefits reported from connecting with peers, including better understanding of experiences and promoting recovery^104^. Pre-to-post reductions on AH severity have been observed^297,298^	Systematic review^104^	No randomised controlled trials on outcomes^104^

*AH* Auditory hallucination.

Promising efficacy was also found for cognitive behavioural therapy (CBT), but primarily derived from intervention studies for psychosis, rather than pertaining to voices/APE. Psychological treatments developed specifically for voice-hearing^199^, including transdiagnostically^202–204^, demonstrate encouraging results, though intervention initiatives for other APE are notably scarce. Latest developments within psychological interventions involve evolution of existing therapies, e.g., refs. ^203,205^, incorporation of technological advances^206^ and highly limited consideration of multisensory hallucinations^207^. Psychological approaches have evolved from initial applications of CBT to incorporate acceptance- and mindfulness-based principles^208^, methods targeting relational dynamics to voice-hearing^209^, and trauma-focused strategies^210,211^. Voice-specific digital innovations primarily comprise smartphone applications, e.g., refs. ^202,212^ and use of virtual/augmented^213^ reality to represent voices in the form of avatars^214^. These latter therapies are still in development, and necessitate fine-tuning and replication.

## Limitations and future directions

### Gaps in existing knowledge

This review has summarised the state of research regarding voices/APE, highlighting prominent gaps in existing knowledge^215^. Owing to the sheer breadth of this literature, systematic synthesis was not possible in the current review, though it is hoped that more specific systematic reviews and targeted empirical works are developed from the findings outlined. Although these findings may be somewhat limited by the selection bias inherent within narrative reviews, our international and diverse authorship and lived experience contributions are intended to represent the field, taking into account a range of cultural, clinical and empirical lenses. There remains much we do not know and need to address, but an overarching message is perhaps one of specificity. Prevailing studies need to move beyond voices in psychosis to inclusively examine hallucinations across a range of sensory modalities (beyond the five dominant senses covered)^216^ as well as in other psychiatric conditions^217^, to address phenomenological, mechanistic and interventional concerns. Translation of existing knowledge can expedite this process, including appropriate cultural adaptations to meet prevailing needs.

### Imminent research priorities

Table 6 provides a comprehensive breakdown by topic of top research priorities for voices/APE identified by the review, accompanied by in-text emphasis on three focal areas. First, improvements to existing assessment tools and overall methodological consistency, in terms of task standardisation and homogenous cut-off scores delineating voices/APE, e.g., refs. ^131^, are warranted. Newer improved measures exploring greater phenomenological detail or lesser studied sensory domains, including multisensory/multimodal, are also needed. Specific facets involving distress (e.g., if/when non-auditory hallucinations confer distress) and valence (e.g., positive voices/APE), pertinent to therapeutic interventions, deserve further investigation. Some interest has been shown in positive voices^218,219^, although this has yet to generalise to other sensory modalities. In turn, methodological consistency would facilitate more robust mechanistic conclusions, and is necessary for improved intra- and interdisciplinary collaborations, which are already starting to transpire, e.g., ref. ^189^.

**Table 6:** Top research priorities regarding voices/APE.

Topic/theme	Priority focus/research questions
Sensory modalities	Which other sensory modalities of hallucinations (e.g., sensed presence(s)) warrant further investigation?
	What important nosological, aetiological and therapeutic insights may be delivered in this process?
Assessment tools	Development and validation of assessment tools for unimodal non-auditory as well as multisensory/multimodal hallucinations in psychosis and beyond (e.g., mood and anxiety)
	Collection of sufficient phenomenological information, including positive/adaptive aspects, within newly developed assessment tools
Prevalence	Large-scale prevalence data (other than for voices in psychosis) remains patchy and needs to be remedied
	What are the prominent demographic and clinical correlates of voices/altered perceptual experiences and implications therein?
Phenomenological characterisation	How do nascent phenomenological findings regarding voices in mood and anxiety conditions inform disorder-specific therapeutic approaches?
	How can we systematically study the impact of dimensional mood and anxiety symptoms on voices/altered perceptual experiences?
	How can the study of positive voices/altered perceptual experiences enrich aetiological models and therapeutic interventions?
Methodological enhancements	Increased replicative efforts involving diverse and improved methodologies (e.g., qualitative) accounting for multidisciplinary observations and the totality of service users’ experiences
	There is a lack of longitudinal research for voices/altered perceptual experiences, especially in mechanistic and intervention studies
Trauma	Does (repeated) trauma experienced in adulthood (versus childhood) manifest differently in terms of voices/altered perceptual experiences?
	Are there unique links between trauma and multisensory/multimodal hallucinations, and what therapeutic lessons can we learn?
Neurocognitive mechanisms	There is a need to standardise common neurocognitive batteries/tasks across the field to facilitate meaningful comparisons
	Replicative mechanistic studies into non-auditory and multisensory/multimodal hallucinations are needed
Aetiological models	How can inclusive biopsychosocial explanatory models be developed for voices/altered perceptual experiences? Is this even possible, or are multiple models (e.g., via subtyping) necessary to account for the noted heterogeneity?
	How can prevailing aetiological models incorporate protective/resilience factors to ensure a balanced viewpoint?
Interventions	How can subtyping research contribute meaningfully to interventions specific to voices/altered perceptual experiences?
	Are modality-specific interventions targeted at distressing hallucinations (beyond voices) necessary? If so, how can these borrow from existing knowledge and what would they look like?
Transcultural considerations	There is a need for appropriate modification of assessment tools and treatment protocols, beyond mere language considerations, to account for cultural differences
	How do we systematically account for transcultural considerations in a way that takes into account the complex and competing demands imposed by social and personal narratives?
	Beyond active lived experience engagement, how can gender and neuro-diverse (and other non-dominant) perspectives be consistently represented in research and clinical practice?

Second, it is imperative to consider new therapeutic applications, especially how relatively effective psychological approaches originating from voices in psychosis may be employed more extensively, in phenomenologically responsive ways^216^, with variations to accommodate modality- and disorder-specific considerations. Early interest in strengths-based approaches, e.g., ref. ^220^ can be renewed and consolidated^129^, such as those involving self-help techniques^104,204,206,221^, peer support^222^, and adaptive coping to voices^221,223^ and images^224^. There may also be scope to borrow from the visual hallucinations sphere pertaining to potential treatments. Successful innovations may be fruitfully incorporated into early intervention protocols, with demonstrated efficacy for first-episode psychosis^225^. Above all, treatment formats should consider direct service user input^129^, be flexible to maximise accessibility, and weigh up the benefits of interventions targeted at specific symptom alleviation versus more holistic recovery-oriented approaches (also see Table 1 for lived experience perspectives).

Third, whilst we have attempted to incorporate transcultural considerations throughout, much remains to be done. To develop better assessment tools, there is a need to surpass mere translation efforts accounting for language differences. A broader question exists of how best to navigate the nuanced intersections of culture, beyond a rudimentary East-West divide, along geographic or national borders, or across dominant religious affiliations. This is especially pertinent in an increasingly globalised world characterised by diminishing homogenised cultural settings^29^. Engaging service user involvement across all levels of research and clinical practice would be ideal, but is imperative for designing and assessing therapeutic interventions^8^. This would comprise negotiating the complexities of what recovery would look like amidst extant personal (e.g., identity) and social (e.g., stigma) challenges. An illustration can be made by considering the narratives of young persons who hear voices^223^, including gender and neuro-diverse perspectives, which so far have been heavily underrepresented^226^. Systemic power imbalances in medical settings remain challenging to overcome, but a partial, short-term remedy could lie in a conscious shift in the way health professionals dialogically and semantically communicate with persons under their care^227,228^.

## Conclusions

There has been a preponderance of voice-hearing research in psychosis, perhaps owing to the centrality of human language in forging personal identity. Nonetheless, it is evident that APE in sensory modalities (beyond voices) exist and operate across a range of psychiatric (and neurological) groups (beyond psychosis). Thus whilst existing knowledge is heavily skewed towards voices in psychosis, tentative conclusions may be drawn by extrapolating from the current literature: (i) vast phenomenological heterogeneity precludes identifiable facets conveying diagnostic relevance, with the exception of distress; (ii) multisensory/multimodal experiences in psychosis (and other psychiatric conditions) represent the norm, with the lack of appropriate assessment tools partly liable for underreporting; (iii) existing mechanistic research needs to be amalgamated across sensory modalities, disorders, and disciplines to yield inclusive explanatory models, also accounting for powerful sociocultural influences; (iv) phenomenological considerations are still important for tailored therapeutic interventions, where experiential subtypes may be matched with effective treatment options; and (v) lived experience perspectives are key to designing and assessing therapies that meet service users’ needs, especially incorporating previously neglected viewpoints (e.g., ‘non-Western’, female, gender or neuro-diverse). There is a clear and inclusive way to advance the field of voices/APE so that scientific gains may be put towards alleviation of distress (when experienced), regardless of sensory modality or diagnostic condition.

## Supplementary information

Supplementary materials

## Data Availability

N/A
