# Supplementary material for: Hearing voices and other altered perceptual experiences across psychotic, mood, and anxiety disorders: from phenomenology and mechanisms to future directions
Source: Schizophrenia (Heidelb). 2025 Sep 29;11(1):121. doi: 10.1038/s41537-025-00673-3 (PMC12480513; doi:10.1038/s41537-025-00673-3)
Supplement: Supplementary file 1 — Supplementary materials [file 41537_2025_673_MOESM1_ESM.pdf]

Table A. Structure and process of lived experience focus group sessions.

Session	Aim	Content	Discussion points
All	Housekeeping/ ground rules	Preferred style of communication, options regarding mode of feedback and privacy/confidentiality	*Beginning of session:* Panel reminded:<br>i) based on personal preference, cameras can be on or off, verbal or chat function can be used<br>ii) option to stay back after the session to share questions/comments, and email contact anytime is welcome;<br>iii) any expertise the panel is willing to share is welcomed and valued<br>iv) previous session recap with extra thoughts, comments, questions invited, and session content/structure introduced<br>*End of session:* Proposed structure of next session, with participants asked for feedback to facilitate involvement
1	Orientation	Introductions	Facilitators introduced the project and research group; panel members introduced themselves
		Overview of project	Structure and content of main report explained; general feedback and open discussion regarding initial impressions and important points to include
		Lived experience contributions	Proposed outline: Overall structure approved; phenomenology: To focus on heterogenous and individual experience; cultural perspectives: To discuss nuanced and subcultural influences, in addition to broader cultural factors
2	Brainstorm	Terminology	Use of language by clinicians, researchers, peer support groups and self
		Clinical assessment tools	Prior experiences, and aspects of voices/altered perceptual experiences that were important or neglected
		Phenomenology	Misunderstood, misrepresented, or underrepresented aspects of voices/altered perceptual experiences
		Biopsychosocial mechanisms	Factors or stressors which may have contributed to voices/altered perceptual experiences
		Treatments	Which are more or less helpful? What do they lack or should look to incorporate?
		Transcultural considerations	What aspects of culture and subculture might change a person's voices/altered perceptual experiences?
		Future directions	What can/should we do better as researchers and clinicians?
		Lived experience contributions	Terminology: To favour "voices/altered perceptual experiences" over "hallucinations"; assessment tools: To point out lack of nuance in available measures; phenomenology: To emphasise the influence of mood and anxiety; biopsychosocial: To highlight the areas of trauma, mood, and adolescence; treatments: To include consideration of holistic (and multimodal) therapies; future directions: To discuss how to improve therapeutic interactions with clinicians, and consider bias (e.g. technological) in treatment innovations
3	Interim report feedback	Prior to session	Report draft sent out, with feedback invited before, during or after session
		Introduction	Terminology, scope, aims and research questions, Table 1 and Table A
		Phenomenology	Clinical assessment tools, prevalence, phenomenology, transcultural research, Table 3 and Figure 1
		Biopsychosocial mechanisms	Trauma, neurocognition, other risk and protective factors

		Models and interventions	Methods of subtyping, etiological models and voice-specific interventions and Table 5
		Future directions	Future research priorities, conclusions and Table 6
		General feedback	What did you like/not like? Was any content missing, unnecessary, not informative or need to be managed differently?
		Lived experience contributions	Main report: Overall structure and content approved; treatments: To highlight side effects of antipsychotic medications, and need for patient involvement in peer support and therapeutic groups; future directions: To mention holistic consideration of the individual and emphasise importance of lived experience perspectives in research; tables and figures: Specific minor comments to improve inclusiveness and readability
4	Final report feedback and other deliverables	Main report	Structure and overview of report summated any extra feedback welcomed
		Other deliverables	One page summary, oral presentation, infographic and slide pack shared and discussed
		Lived experience contributions	Other deliverables: Guided presentation of lay and infographic summaries, video and slide pack, accompanied by in-depth discussion of numerous, minor language and presentation tweaks to improve inclusiveness and readability

Note: Lived experience focus group were recruited via a call for interest through the patient and public involvement networks of individual team members. Five individuals across Australia, India and the United Kingdom, with a range of diagnoses and voices/altered perceptual experiences and varied gender identities self-selected and were included in the focus group. PPI attendance rate was 100% during all sessions.

Table B. Summary of prevailing models underlying voices/APE.

Name of model, citation	Field	Modality	Description	Constructive critique
Cognitive				
Heightened salience of inner speech [181,182]	Psychology	AH	Proposes heightened salience of inner speech in voice-hearers leads to its misattribution to an external source (i.e., externalising biases)	Meta-analytic support of an externalising bias in voice-hearers with SSD [135], associated with trauma, and coupled with AH severity [181,310]. However, this has been suggested to represent a general deficit in associative memory processes in SSD [311,312]; not reliably replicated in non-clinical voice-hearers [313-316]. Little meta-analytic support from the AH neuroimaging literature [181], some support from VH PD literature [317], however the role of this model in multimodal PE has been challenged [6]
Intentional inhibition/ contextual memory [179,180]	Psychology	AH	Suggests AH are auditory representations derived from unintentional activation/deficits in intentional inhibition of memory and other irrelevant mental associations. Having lost contextual cues, such memories are not recognised, leading to AH	Robust support for impaired intentional inhibition in voice-hearers with SSD [130], coupled with AH severity [179]. Replicated in non-clinical voice-hearers [130], and associated with hallucination proneness [318]. AH, OH and VH source memory deficits not reliably replicated in non-clinical populations [319], may be specific to clinical cohorts [6,320]; this discrepancy has been supported by neuroimaging findings [312]
Cortical				
Excitatory/inhibitory imbalance [183]	Psychology, Neurology	AH, VH	Draws on cognitive, computational, molecular, pharmacological, functional neuroimaging and neurostimulation studies, to posit that an imbalance between excitatory and inhibitory cortical mechanisms contributes to AH and VH	Evidence of an excitatory/inhibitory imbalance in AH/VH in SSD [183], VH in AD [321], PE in PTSD [191]. Non-clinical studies suggest excitatory/inhibitory imbalances may be associated with AH, but preserved in VH [322]; model yet to be applied to GH, OH or S/TH
Interhemispheric miscommunication [184]	Psychology	AH	Suggests deviations from a healthy level of connectivity between the A1 homologues contribute to AH. Involves reduced left-sided lateralisation of temporal language processing in the cortex [323], in conjunction with overweighted top-down processes and impaired intentional inhibition [184]	Evidence of A1 hyperconnectivity in non-clinical and first-episode psychosis voice-hearers, which develops into A1 hypoconnectivity with chronicity in clinical samples [324]. Interhemispheric A1 hypoconnectivity in voice-hearers confirmed with meta-analysis [325], which decreases when an individual is actively hearing voices [326]. Functional and structural neuroimaging evidence in clinical [327-329] and non-clinical voice-hearers [330,331]. Applicability may be limited to AH
Trauma-related				
Adverse life events and phenomenology [188]	Psychology	AH	Proposes adverse life events, mediated by factors including altered emotional processing, dissociation, hypervigilance, perceived social rank, self-blame and shame underscore negative voice-content. Draws on epidemiological, phenomenological and neuroimaging evidence, and considers transcultural perspectives	Correlations between trauma and general [188] or negative voice content in DID, PTSD, SSD [89,332]; some evidence to the contrary [333]. Heightened limbic activity seen during resting state in voice-hearers with SSD [334], in response to emotional speech [131], and in the non-clinical population [335]. Higher likelihood of positive voice content in cultures where voices/altered perceptual experiences are less stigmatised [14,15]. Further support from predictive coding account of PE in PTSD [191]. Model yet to consider the role of genetics, or provide explanations for AH in voice-hearers without trauma history; also yet to be tested in neurology or PE outside AH

Name of model, citation	Field	Modality	Description	Constructive critique
Childhood trauma and neurodevelopment [111,112]	Psychology		Integrates cognitive, epidemiological and neurobiological evidence to propose a childhood trauma phenotype of psychosis. Posits a genetic vulnerability, alongside adverse events in childhood, lead to affective challenges, structural and functional cortical alterations and ultimately psychosis	Model considers psychosis generally (i.e., non-specific), with evidence of its contribution to AH (e.g., dose-dependent relationship between childhood trauma and AH in SSD [336] and general population [337]). Relationship between childhood sexual abuse and AH confirmed with systematic review [114]. Neurobiological support of white matter microstructural changes [338], altered brain derived neurotrophic factor levels [339]. No evidence of hypothalamic-pituitary-adrenal axis dysfunction in AH. Model yet to consider other diagnoses
Integrated models				
Integrated cognitive model for multimodal hallucination [6]	Psychology, Neurology	Multi-modal	Builds on the modality-general and modality-specific processes outlined by [145] to propose a framework which may explain multimodal hallucination. Draws on evidence from misattribution biases, predictive coding and circular inference, reality monitoring and social cognition, predominantly in SSD and PD	Sensory processing impairments in AH [325], VH [340] and OH [341], with associated neurological alterations confirmed in AH [131] and VH [132], support modality-specific processes. Predictive coding and circular inference models as modality-general processes supported by cognitive and neurological evidence in AH/VH in AD, PD, and SSD [183], neuro-philosophical [342] and computational work in SSD [343], and considerable work in PTSD [134,191]. Poses challenges for the role of misattribution biases and reality monitoring in modality-general processes. Role of social cognition requires further research. Model yet to be verified in other cohorts, or integrate GH or S/TH
Integrated cortical models for transdiagnostic hallucinations [132,190]	Psychology, Neurology	AH, VH	Meta-analytic evidence from structural [132] and functional [344] neuroimaging investigations of transdiagnostic AH/VH	Structural: Differential patterns of grey matter and cortical thickness reductions between PD and SSD; (more related to auditory processing in SSD, and visual processing in PD). Correlations between grey matter reductions and AH found in SSD [132]. However, PE chronicity may influence structural changes [324]. Field would benefit from further investigations, with only eight studies per clinical arm for comparative aspect of meta-analysis<br>Functional: Similar to structural evidence [132], functional alterations appear specific to modality of PE; both offer support for modality-specific processes previously outlined [6,145]. Supported by evidence from neurophysiological investigations in AH [131,345]. Analyses limited by high prevalence of region of interest studies and small number of VH studies
Visual hallucinations as veridical perceptions [189]	Neurology	VH	Draws on eight models (e.g., Bayesian models [346], deafferentation [347], reality monitoring [348], perception and attention [349], functional network decoupling [350-352]) to posit an overarching framework for complex VH. Addresses disparities between these models and suggests an interplay of cognitive/emotional states and visual input altering expectancy and perception via attention, inference and sensory processing	Framework is structured on the subjective likeness of VH and veridical visual perception; some support from a small base of VH symptom capture studies with functional neuroimaging, which indicate associative, but not necessarily primary, visual cortex involvement [350,353,354]. May support modality-specific processes [6,145], although model yet to be applied to simple VH<br>Opposition to the veridical account of PE seen in AH literature, where many proponents state that voices are not akin to veridical sensory experiences [25,355,356].<br>Ultimately, authors have proposed the framework as a means to test its validity, with numerous caveats and research questions outlined [189]. Investigations into treatments which target different components of the framework may offer insights into its true synergy and therapeutic utility

Note: Unimodal S/TH, OH and GH models are missing from the extant literature. A1: Primary auditory cortex; AD: Alzheimer's disease; AH: Auditory hallucination; DID: Dissociative identity disorder; GH: Gustatory hallucination; OH: Olfactory hallucination; PD: Parkinson's disease; PE: Voices/altered perceptual experiences; PTSD: Post-traumatic stress disorder; S/TH: Somatic-tactile hallucination; SSD: Schizophrenia spectrum disorders; VH: Visual hallucination.

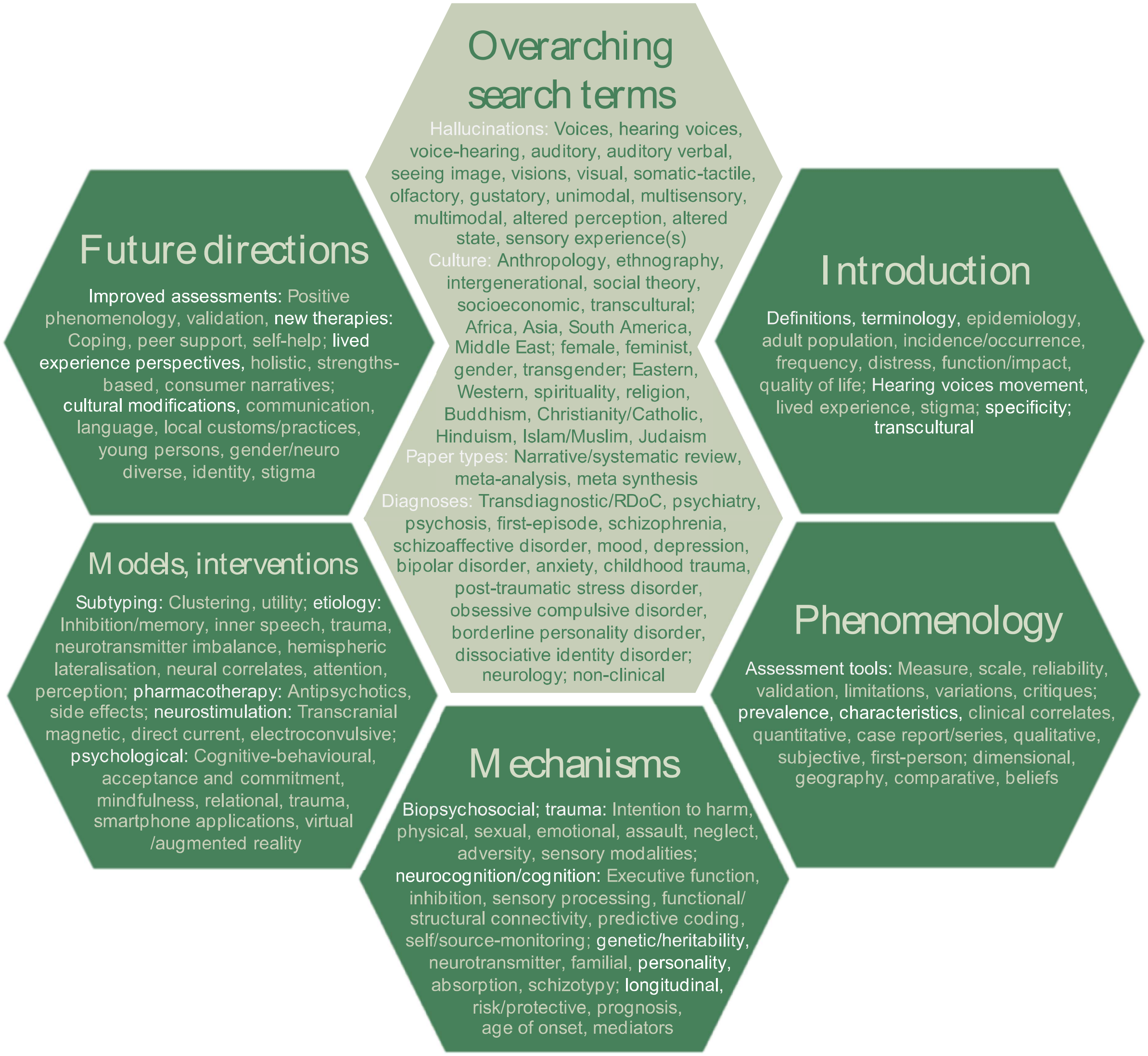

Figure A: Search methodology mind map. Overarching search terms (in beige hexagons) were used in conjunction with individual search terms for each research question (in green hexagons) to conduct a structured, though not exhaustive literature searches.

310 Steel, C., Fowler, D. & Holmes, E. A. Trauma-related intrusions and psychosis: an information processing account. *Behavioural and Cognitive Psychotherapy* **33**, 139-152 (2005).

311 Achim, A. M. & Weiss, A. P. No evidence for a differential deficit of reality monitoring in schizophrenia: A meta-analysis of the associative memory literature. *Cognitive Neuropsychiatry* **13**, 369-384, doi:10.1080/13546800802299476 (2008).

312 Badcock, J. C. & Hugdahl, K. Cognitive mechanisms of auditory verbal hallucinations in psychotic and non-psychotic groups. *Neuroscience & Biobehavioral Reviews* **36**, 431-438, doi:https://doi.org/10.1016/j.neubiorev.2011.07.010 (2012).

313 Garrison, J. R. *et al.* Testing continuum models of psychosis: No reduction in source monitoring ability in healthy individuals prone to auditory hallucinations. *Cortex* **91**, 197-207, doi:https://doi.org/10.1016/j.cortex.2016.11.011 (2017).

314 Larøi, F., Van der Linden, M. & Marczewski, P. The effects of emotional salience, cognitive effort and meta-cognitive beliefs on a reality monitoring task in hallucination-prone subjects. *British Journal of Clinical Psychology* **43**, 221-233 (2004).

315 McKague, M., McAnally, K. I., Skovron, M., Bendall, S. & Jackson, H. J. Source monitoring and proneness to auditory-verbal hallucinations: a signal detection analysis. *Cogn Neuropsychiatry* **17**, 544-562, doi:10.1080/13546805.2012.676311 (2012).

316 Moseley, P. *et al.* Continuities and discontinuities in the cognitive mechanisms associated with clinical and non-clinical auditory verbal hallucinations. (2020).

317 Shine, J. M. *et al.* Imagine that: elevated sensory strength of mental imagery in individuals with Parkinson's disease and visual hallucinations. *Proc Biol Sci* **282**, 20142047, doi:10.1098/rspb.2014.2047 (2015).

318 Vercammen, A. & Aleman, A. Semantic expectations can induce false perceptions in hallucination-prone individuals. *Schizophr Bull* **36**, 151-156, doi:10.1093/schbul/sbn063 (2010).

319 Moseley, P. *et al.* Continuities and Discontinuities in the Cognitive Mechanisms Associated With Clinical and Nonclinical Auditory Verbal Hallucinations. *Clinical Psychological Science* **10**, 752-766, doi:10.1177/21677026211059802 (2022).

320 Diederich, N. J., Goetz, C. G. & Stebbins, G. T. Repeated visual hallucinations in Parkinson's disease as disturbed external/internal perceptions: focused review and a new integrative model. *Movement disorders: official journal of the Movement Disorder Society* **20**, 130-140 (2005).

321 El Haj, M. *et al.* Clinical and neurocognitive aspects of hallucinations in Alzheimer's disease. *Neuroscience & Biobehavioral Reviews* **83**, 713-720, doi:https://doi.org/10.1016/j.neubiorev.2017.02.021 (2017).

322 de Boer, J. N. *et al.* Auditory hallucinations, top-down processing and language perception: a general population study. *Psychological Medicine* **49**, 2772-2780, doi:10.1017/S003329171800380X (2019).

323 Hugdahl, K. *et al.* Auditory verbal hallucinations in schizophrenia as aberrant lateralized speech perception: evidence from dichotic listening. *Schizophrenia research* **140**, 59-64 (2012).

324 Ćurčić-Blake, B. *et al.* Interaction of language, auditory and memory brain networks in auditory verbal hallucinations. *Progress in neurobiology* **148**, 1-20 (2017).

325 Ocklenburg, S., Westerhausen, R., Hirnstein, M. & Hugdahl, K. Auditory hallucinations and reduced language lateralization in schizophrenia: a meta-analysis of dichotic listening studies. *Journal of the International Neuropsychological Society: JINS* **19**, 410 (2013).

326 Løberg, E.-M., Jørgensen, H. A. & Hugdahl, K. Dichotic listening in schizophrenic patients: effects of previous vs. ongoing auditory hallucinations. *Psychiatry research* **128**, 167-174 (2004).

327 Gavrilescu, M. *et al.* Reduced connectivity of the auditory cortex in patients with auditory hallucinations: a resting state functional magnetic resonance imaging study. *Psychological medicine* **40**, 1149, doi:10.1017/S0033291709991632 (2010).

328 Briend, F. *et al.* Impact of rTMS on functional connectivity within the language network in schizophrenia patients with auditory hallucinations. *Schizophrenia research* **189**, 142-145, doi:https://doi.org/10.1016/j.schres.2017.01.049 (2017).

329 Henshall, K. R. *et al.* Interhemispheric transfer time in patients with auditory hallucinations: an auditory event-related potential study. *International journal of psychophysiology* **84**, 130-139, doi:10.1016/j.ijpsycho.2012.01.020 (2012).

330 Tagliazucchi, E., Von Wegner, F., Morzelewski, A., Brodbeck, V. & Laufs, H. Dynamic BOLD functional connectivity in humans and its electrophysiological correlates. *Frontiers in Human Neuroscience* **6**, doi:10.3389/fnhum.2012.00339 (2012).

331 Thiebes, S. *et al.* Alterations in interhemispheric gamma-band connectivity are related to the emergence of auditory verbal hallucinations in healthy subjects during NMDA-receptor blockade. *Neuropsychopharmacology* **43**, 1608-1615, doi:10.1038/s41386-018-0014-z (2018).

332 Scott, M., Rossell, S. L., Meyer, D., Toh, W. L. & Thomas, N. Childhood trauma, attachment and negative schemas in relation to negative auditory verbal hallucination (AVH) content. *Psychiatry research* **290**, 112997 (2020).

333 Næss, J. Ø., Hirnstein, M., Kusztrits, I. & Larøi, F. An online survey on clinical and healthy individuals with auditory verbal hallucinations: Abuse did not lead to more negative voice content. *Schizophrenia Research*, doi:https://doi.org/10.1016/j.schres.2022.11.020 (2022).

334 Alderson-Day, B. *et al.* Auditory Hallucinations and the Brain's Resting-State Networks: Findings and Methodological Observations. *Schizophrenia Bulletin* **42**, 1110-1123, doi:10.1093/schbul/sbw078 (2016).

335 van Lutterveld, R., Diederen, K. M. J., Otte, W. M. & Sommer, I. E. Network analysis of auditory hallucinations in nonpsychotic individuals. *Human Brain Mapping* **35**, 1436-1445 (2014).

336 Shevlin, M., Dorahy, M. & Adamson, G. Childhood traumas and hallucinations: an analysis of the National Comorbidity Survey. *Journal of psychiatric research* **41**, 222-228 (2007).

337 Whitfield, C. L., Dube, S. R., Felitti, V. J. & Anda, R. F. Adverse childhood experiences and hallucinations. *Child Abuse Negl* **29**, 797-810, doi:10.1016/j.chiabu.2005.01.004 (2005).

338 Sato, Y. *et al.* Relationship Between White Matter Microstructure and Hallucination Severity in the Early Stages of Psychosis: A Diffusion Tensor Imaging Study. *Schizophrenia Bulletin Open* **2**, sgab015, doi:10.1093/schizbullopen/sgab015 (2021).

339 Li, X. *et al.* Metabolomics and Cytokine Analysis for Identification of Schizophrenia with Auditory Hallucination. *Clinical and Investigative Medicine* **45**, E39-48 (2022).

340 Montagnese, M. *et al.* Cognition, hallucination severity and hallucination-specific insight in neurodegenerative disorders and eye disease. *Cognitive Neuropsychiatry* **27**, 105-121, doi:10.1080/13546805.2021.1960812 (2022).

341 McAuley, J. H. & Gregory, S. Prevalence and clinical course of olfactory hallucinations in idiopathic Parkinson's disease. *J. Parkinsons Dis.* **2**, 199-205, doi:10.3233/JPD-2012-012086 (2012).

342 Rappe, S. & Wilkinson, S. Counterfactual cognition and psychosis: adding complexity to predictive processing accounts. *Philosophical Psychology* **36**, 356-379 (2023).

343 Fletcher, P. C. & Frith, C. D. Perceiving is believing: a Bayesian approach to explaining the positive symptoms of schizophrenia. *Nature Reviews Neuroscience* **10**, 48-58 (2009).

344 Zmigrod, L., Garrison, J. R., Carr, J. & Simons, J. S. The neural mechanisms of hallucinations: A quantitative meta-analysis of neuroimaging studies. *Neuroscience & Biobehavioral Reviews* **69**, 113-123, doi:https://doi.org/10.1016/j.neubiorev.2016.05.037 (2016).

345 Arora, M., Knott, V. J., Labelle, A. & Fisher, D. J. Alterations of Resting EEG in Hallucinating and Nonhallucinating Schizophrenia Patients. *Clinical EEG and Neuroscience* **52**, 159-167, doi:10.1177/1550059420965385 (2020).

346 Friston, K. J. Hallucinations and perceptual inference. *Behavioral and Brain Sciences* **28**, 764-766, doi:10.1017/S0140525X05290131 (2005).

347 Burke, W. The neural basis of Charles Bonnet hallucinations: a hypothesis. *Journal of Neurology, Neurosurgery & Psychiatry* **73**, 535-541 (2002).

348 Barnes, J., Boubert, L., Harris, J., Lee, A. & David, A. S. Reality monitoring and visual hallucinations in Parkinson's disease. *Neuropsychologia* **41**, 565-574 (2003).

349 Collerton, D., Perry, E. & McKeith, I. Why people see things that are not there: a novel perception and attention deficit model for recurrent complex visual hallucinations. *Behavioral and Brain Sciences* **28**, 737-757 (2005).

350 Ffytche, D. H. The hodology of hallucinations. *Cortex* **44**, 1067-1083 (2008).

351 Onofrj, M., Espay, A. J., Bonanni, L., Delli Pizzi, S. & Sensi, S. L. Hallucinations, somatic-functional disorders of PD-DLB as expressions of thalamic dysfunction. *Movement Disorders* **34**, 1100-1111 (2019).

352 Shine, J. M., Halliday, G. M., Naismith, S. L. & Lewis, S. J. G. Vol. 26 2154-2159 (Wiley Online Library, 2011).

353 van Ommen, M. M., van Laar, T., Renken, R., Cornelissen, F. W. & Bruggeman, R. Visual Hallucinations in Psychosis: The Curious Absence of the Primary Visual Cortex. *Schizophrenia Bulletin* **49**, S68-S81, doi:10.1093/schbul/sbac140 (2023).

354 Goetz, C. G., Vaughan, C. L., Goldman, J. G. & Stebbins, G. T. I finally see what you see: Parkinson's disease visual hallucinations captured with functional neuroimaging. *Movement Disorders* **29**, 115-117, doi:https://doi.org/10.1002/mds.25554 (2014).

355 Moritz, S. & Larøi, F. Differences and similarities in the sensory and cognitive signatures of voice-hearing, intrusions and thoughts. *Schizophrenia research* **102**, 96-107 (2008).

356 Bleuler, E. Dementia praecox or the group of schizophrenias. (1950).
